# An Observational Study of Fungal Infections in COVID-19: Highlighting the Role of Mucormycosis in Tertiary Healthcare Settings

**DOI:** 10.7759/cureus.57295

**Published:** 2024-03-30

**Authors:** Rajender Singh, Garima Mittal, Barnali Kakati, Nupur Koul

**Affiliations:** 1 Microbiology, Himalayan Institute of Medical Sciences, Swami Rama Himalayan University, Dehradun, IND

**Keywords:** fungal infection, covid-19-associated mucormycosis, diabetes, rhizopus, medical comorbidities

## Abstract

Background

Fungal infections, especially mucormycosis, have remarkably surged during the coronavirus disease 2019 (COVID-19) era, especially during the second wave peak of the pandemic raising the concern of the clinicians for the admitted patients. Steroid therapy, diabetes, and other immunocompromised states are more commonly associated with COVID-19-associated mucormycosis (CAM).

Aim and objective

The aim of this study is to ascertain the prevalence of fungal infections amidst the second wave of the COVID-19 pandemic and discern the associated risk factors.

Materials and methods

During the second peak of COVID-19, samples were received in the microbiology laboratory from all clinically suspected mucormycosis patients. These samples underwent processing for potassium hydroxide (KOH) wet mount, fungal culture on Sabouraud's dextrose agar (SDA) medium, and COVID-19 reverse transcription-polymerase chain reaction (RT-PCR) testing. All relevant clinical and associated risk factors were tabulated and analyzed.

Results

Among the 107 suspected cases of mucormycosis, 39 (36.4%) were confirmed positive for COVID-19 via RT-PCR, while 68 (63.6%) tested negative. Males exhibited a predominant infection rate, with the rhinocerebral system being the most commonly affected site. Significantly higher mortality rates were observed in COVID-19-associated mucormycosis (CAM) patients (33.4%) compared to those without COVID-19 (5.9%), with a notable p-value of 0.0005. CAM patients also demonstrated a higher frequency of ICU admissions (77%) compared to non-COVID-19-associated mucormycosis patients (21.4%), a statistically significant finding (p-value of 0.007). Additionally, immunocompromised states, diabetes, and the administration of oxygen therapy were identified as significant risk factors in CAM (p < 0.05). Notably, mucormycosis accounted for the majority of fungal isolates (48.27%) among COVID-19 patients.

Conclusion

Mucormycosis infection is more commonly seen in COVID-19-infected patients as compared to non-COVID-19 patients, especially with comorbidities such as diabetes mellitus, steroid usage, and other immunocompromised states.

## Introduction

The coronavirus disease 2019 (COVID-19) pandemic has led to a global increase in severe acute respiratory syndrome coronavirus 2 (SARS-CoV-2) cases, prompting the need for whole genomic sequencing and epidemiological studies to understand the severity of infections caused by emerging variants of concern [1]. Scientists and diagnosticians have been urgently investigating the causes of the increased transmissibility of these strains to help control the surge in cases and alleviate human suffering [2]. While battling the challenges of the pandemic's second wave, a concerning rise in an invasive and potentially life-threatening infection known as "black fungus," caused by the Mucorales family, has raised global alarm [3]. In India, during the peak dominance of the Delta variant B.1.617.2, around 187 cases of coronavirus disease 2019-associated mucormycosis (CAM) were reported from April to June 2021 [4]. Until June 2021, India witnessed a record of approximately 28,252 cases of mucormycosis, with 24,370 cases having a history of COVID-19 and around 17,601 cases reporting a history of diabetes [5]. The highest number of mucormycosis cases recorded was 6,329 in Maharashtra state [6].

The term "mucormycosis" was coined by Baker in 1957 [7]. The filamentous forms of these fungi can easily evade a weakened immune system, leading to infections. The infection can be transmitted through the inhalation of fungal spores or the inoculation of the fungi into cuts or abrasions [8]. Mucormycosis can be classified into six types based on the location of occurrence, with the most common being rhino-orbital cerebral mucormycosis (ROCM), followed by cutaneous, pulmonary, gastrointestinal, disseminated, and uncommon sites [9].

While the commonly associated risk factors for CAM are underlying immunosuppressive conditions such as uncontrolled diabetes mellitus, prolonged corticosteroid usage, organ transplant, and hematological conditions, COVID-19 infection itself can act as a trigger for mucormycosis [10]. Other risk factors studied and reported include prolonged hospitalization, oxygen therapy, antibiotic use, preexisting paranasal diseases, and cytokine storm [11,12].

In light of this background, a prospective observational study was conducted in the Department of Microbiology during the second wave of the pandemic to determine the prevalence of mucormycosis among COVID-19 patients and identify associated risk factors.

## Materials and methods

Study design

This observational cross-sectional study was conducted at the Department of Microbiology, Himalayan Institute of Medical Sciences, Swami Rama Himalayan University, Dehradun. All clinical samples (convenience sampling method) received in the laboratory over three months from April 15 to June 15, 2021, were processed microbiologically as standard operating procedure (SOP). The primary aim was to investigate and diagnose cases of mucormycosis in patients admitted to the hospital during this second COVID-19 wave.

Inclusion criteria

The inclusion criteria included clinically suspected cases of mucormycosis, recent COVID-19-suspected patients of all age groups, both genders, and those admitted to the hospital during the specified study duration.

Exclusion criteria

The exclusion criteria included patients with previous COVID-19 infection, patients already undergoing treatment for mucormycosis at the time of admission, and non-consenting patients.

Ethical approval

The study received approval from the Research Committee of the Himalayan Institute of Medical Sciences with approval number HIMS/RC/2021/184. This ensured that the research adhered to ethical standards and guidelines.

Sample collection

Various samples were collected from suspected mucormycosis cases, including nasal crust/tissue biopsy, nasal swabs, mucopus, and sputum. The collection process followed standardized protocols to maintain consistency.

Microbiological diagnosis (confirmed cases)

Potassium Hydroxide (KOH) Mount

The samples collected were subjected to a potassium hydroxide (KOH) mount to examine fungal elements. This process aids in the preliminary identification of fungal structures.

Fungal Culture

Samples were cultured on Sabouraud's dextrose agar (SDA) plates. These plates were then incubated at 25 degrees Celsius to encourage fungal growth. 

Fungal Identification

Upon obtaining fungal growth on SDA, identification was performed through a lactophenol cotton blue (LPCB) mount. LPCB staining enhances the visualization of fungal structures, facilitating accurate identification.

Data analysis

The collected data, including demographic information and laboratory results, were analyzed using appropriate statistical methods (MS Excel, Microsoft Corp., Redmond, WA) to conclude the prevalence, p-value, and characteristics of mucormycosis cases during the specified timeframe.

## Results

Throughout the study duration, 107 cases clinically suspected of mucormycosis underwent microbiological evaluation for laboratory confirmation. Among these cases, 39 (36.4%) tested positive for COVID-19, while 68 (63.6%) tested negative. The median age distribution for suspected mucormycosis was approximately 46 years in COVID-19-positive patients and 57 years in COVID-19-negative patients. Males were predominantly affected in both COVID-19-positive (74.35%) and COVID-19-negative (64.70%) suspected mucormycosis cases. Rhinocerebral involvement was the most common site observed in suspected cases, followed by pulmonary involvement in both COVID-19 and non-COVID-19 patients. The mortality rate was significantly higher in COVID-19-associated suspected mucormycosis cases (33.4%) compared to COVID-19-negative cases (5.9%) (p-value of 0.0005). The prevalence of laboratory culture-confirmed mucormycosis cases was 13 (33.3%) among COVID-19-positive patients and 14 (20.6%) among COVID-19-negative patients. COVID-19-associated mucormycosis patients required more frequent ICU admission (77%) compared to non-COVID-19 mucormycosis patients (21.4%), which was statistically significant (p-value of 0.007) (Table 1).

**Table 1:** Frequency of suspected cases of mucormycosis among COVID-19-positive and COVID-19-negative patients COVID-19: coronavirus disease 2019

Clinico-demographic characteristics		COVID-19-positive	COVID-19-negative	Total	P-value
Clinically suspected cases of mucormycosis (n = 107)		39 (36.4%)	68 (63.6%)	107	
Median age		46 years	57 years	51.5	
Sex distribution	Male	29 (74.35%)	44 (64.70%)	73 (68.2%)	0.3020
	Female	10 (25.64%)	24 (35.29%)	34 (31.8%)	
Distribution of clinical type of suspected mucormycosis	Rhinocerebral	30 (76.9%)	40 (58.8%)	70 (65.4%)	
	Pulmonary	6 (15.4%)	23 (33.8%)	29 (27.1%)	
	Disseminated	2 (5.1%)	5 (7.4%)	7 (6.5%)	
	Cutaneous	1 (2.6%)	0	1 (0.9%)	
Mortality among suspected mucormycosis	Alive	26 (66.6%)	64 (94.1%)	90 (84.1%)	0.0005
	Died	13 (33.4%)	4 (5.9%)	17 (15.9%)	
Confirmed cases of mucormycosis		13 (33.3%)	14 (20.6%)	27 (25.2%)	
Distribution of confirmed cases of mucormycosis in the ward and ICU	ICU	10 (77%)	3 (21.45%)	13 (48.1%)	0.007
	Ward	3 (23%)	11 (78.6%)	14 (51.9%)	

**Figure 1:**
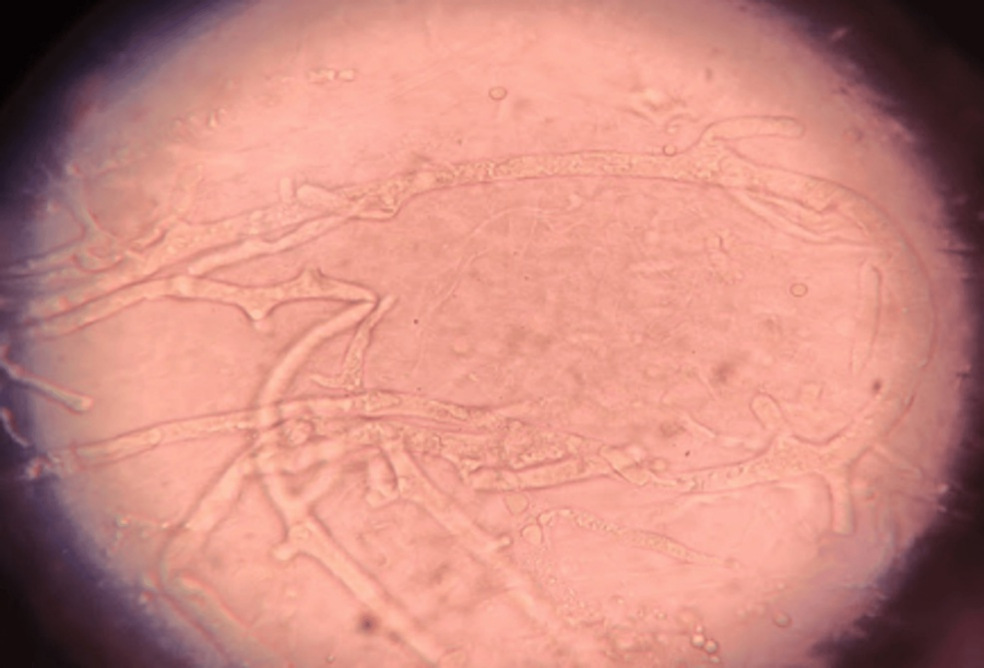
Broad aseptate fungal hyphae in KOH wet mount of nasal crust specimen KOH: potassium hydroxide

**Figure 2:**
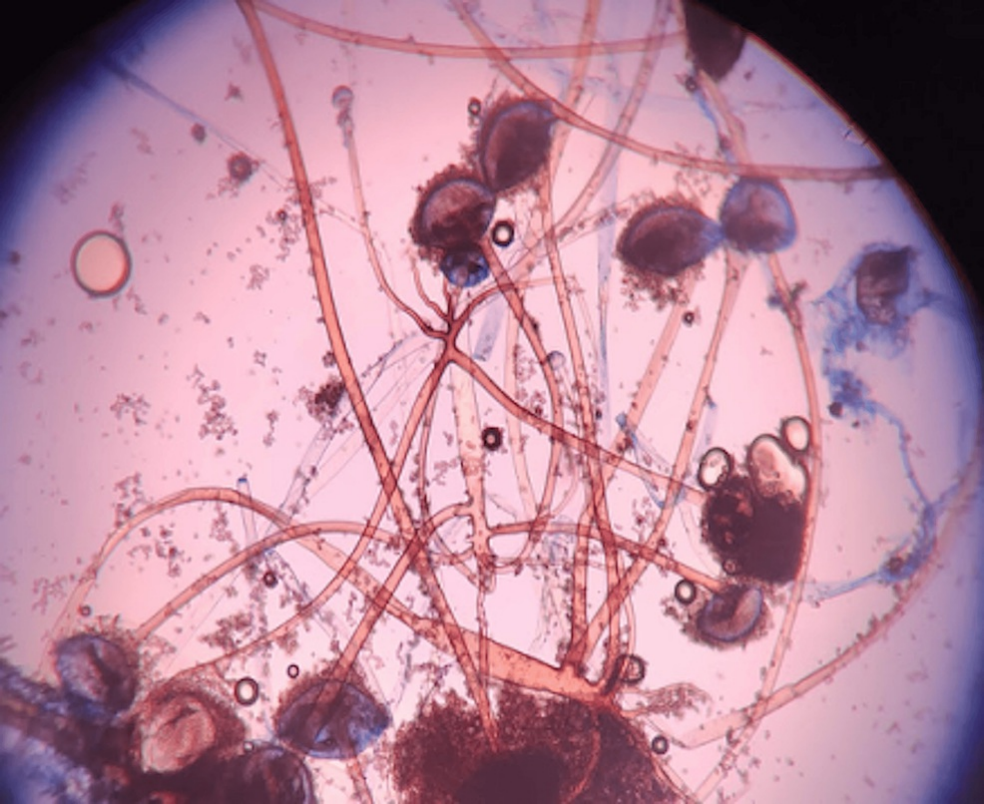
Rhizoids, sporangiophore, and columella of Rhizopus species in LPCB mount LPCB: lactophenol cotton blue

The microscopic evaluation of KOH mounts showed broad aseptate fungal hyphae (Figure 1), and lactophenol cotton blue mounts from the growth of fungal isolates confirmed the presence of *Rhizopus* species (Figure 2).

COVID-19-positive mucormycosis showed statistically significant associations with immunocompromised states of the patients (p-value = 0.0472, Fisher exact test), diabetes (p-value = 0.0213), and the administration of oxygen therapy (p-value = 0.0183) (Table 2).

**Table 2:** Association between the COVID-19 status of mucormycosis cases and various risk factors COVID-19: coronavirus disease 2019

	Immunocompromised	Diabetes	Other comorbidities	Oxygen therapy	Steroid therapy
COVID-19-positive mucormycosis (n = 13)	10 (77%)	9 (69.2%)	4 (31%)	8 (62%)	7 (54.5%)
COVID-19-negative mucormycosis (n = 14)	4 (28.6%)	3 (21.4%)	4 (28.6%)	2 (14.3%)	0
P-value	0.0472	0.0213	0.9	0.0183	0.001

In 41% of suspected mucormycosis cases, other pathogenic fungi were isolated, while 25.6% were culture-negative. Among the COVID-19-negative suspected cases, 41.2% had other pathogenic fungi isolated, and 38.2% were culture-negative. No statistically significant association was observed between mucormycosis and COVID-19 infection (p-value = 0.168) (Table 3).

**Table 3:** Mucormycosis association among COVID-19-positive and COVID-19-negative cases COVID-19: coronavirus disease 2019

	COVID-19-positive (n = 39)	COVID-19-negative (n = 68)	P-value
Mucormycosis	13 (33.3%)	14 (20.6%)	0.1689
Other fungal infections	16 (41%)	28 (41.2%)	
Culture-negative	10 (25.7%)	26 (38.2%)	

KOH wet mounts showed 89.74% sensitivity, while culture showed 74.35% sensitivity in clinically suspected mucormycosis patients. Among all the clinical samples received, mucormycosis accounted for 48.27% of the fungal isolates in COVID-19 patients, followed by *Alternaria* sp. (17.24%). Among mucormycosis patients, *Mucor* species (57%) were the most frequently isolated fungi in COVID-19-positive cases (Table 4).

**Table 4:** Sample-wise distribution of fungal isolates recovered from suspected mucormycosis in COVID-19-positive patients (n = 39) BAFH, broad aseptate fungal hyphae; SFH, septate fungal hyphae; NFES, No fungal element seen; KOH, potassium hydroxide; COVID-19, coronavirus disease 2019

Sample (n = 39)	KOH findings	Frequency	Growth recovered on fungal culture	Number of isolates	
Nasal crust (n = 17)	BAFH	14	Rhizopus species	6 (20.6%)	
			Mucor species	4 (13.8%)	
			Alternaria species	2 (6.9%)	
	SFH	1			
			Fusarium species	2 (6.9%)	
			Trichophyton species	1 (3.4%)	
	NFES	2			
			No fungal growth	2 (5.1%)	
Nasal swab (n = 20)	BAFH	5	Mucor species	3 (10.3%)	
			Alternaria species	3 (10.3%)	
			Candida species	2 (6.9%)	
			Fusarium species	2 (6.9%)	
			Cladosporium species	2 (6.9%)	
	SFH	3	Aspergillus species	1 (3.4%)	
	NFES	12	No fungal growth	7 (17.9%)	
Mucopus (n = 01)	BAFH	1	Mucor species	1 (3.4%)	
Sputum (n = 01)	SFH	1	No fungal growth	1 (2.5%)	
Total (n = 39)	KOH+	25 (64.1%)	Fungal growth+	29 (74.35%)	

The rate of secondary bacterial infection was higher in COVID-19-associated mucormycosis (48.2%) compared to non-COVID-19-associated mucormycosis (21.9%), and a statistically significant association was observed between bacterial coinfection and fungal-infected COVID-19 patients (p-value = 0.0209) (Table 5).

**Table 5:** Bacterial coinfection association among COVID-19 and non-COVID-19 mycosis patient COVID-19: coronavirus disease 2019

	Bacterial coinfection (present)	Bacterial coinfection (absent)
COVID-19-associated mycosis (n = 29)	14 (48.2%)	15 (51.8%)
Non-COVID-19 mycosis (n = 41)	9 (22%)	32 (78%)
P-value = 0.0209		

## Discussion

The sudden increase in mucormycosis cases during the COVID-19 pandemic has attracted the attention of researchers worldwide. In this study, the median age of suspected mucormycosis patients with COVID-19 was relatively lower (46 years) compared to non-COVID-19 patients (57 years), which is consistent with other studies [13-15]. Males predominated in suspected mucormycosis cases, similar to previous studies reporting male predominance [14-21].

Diabetes (69.2%), oxygen therapy (62%), and steroid therapy (54.55%) were found to be statistically significant risk factors associated with COVID-19 infection compared to non-COVID-19 patients. These findings are consistent with previous studies that identified diabetes mellitus as a major comorbidity [13,18,19]. The lack of a statistically significant association between mucormycosis and COVID-19 infection in our study might be due to the inclusion of acute COVID-19 infections confirmed by reverse transcription-polymerase chain reaction (RT-PCR), rather than previous infections with raised antibody titers as seen in other studies [20].

Mucormycosis, caused by various fungal infections within the order Mucorales and the family Mucoraceae, has been observed during the COVID-19 pandemic. In our study, *Mucor* species (27.5%), followed by *Rhizopus* species (20.6%), were the most frequently isolated fungi among COVID-19-positive patients, while other studies reported *Rhizopus* as the most common fungal isolate [19]. These fungi are typically nonpathogenic to immunocompetent individuals but can cause severe infections in immunosuppressed patients with risk factors such as diabetes, steroid intake, and secondary infections. Immune dysfunction, including decreased cluster of differentiation (CD) 4 T cells, CD8 T cells, lymphocytes, delayed interferon (IFN)-gamma response, and prolonged hyperinflammatory states, exacerbates the cytokine storm and promotes the severity of COVID-19 infections [22]. Impaired macrophages and neutrophils' ability to kill fungal hyphae, along with increased fungal heme oxygenase enzyme-promoting iron absorption required for fungal metabolism, contribute to severe infections, particularly in patients with uncontrolled diabetes [22,23].

Rhinocerebral involvement is the most commonly observed clinical form of mucormycosis, characterized by black necrotic eschar formation on nasal turbinates or palates [24].

The immune dysregulation mechanism such as decreased T lymphocytes, CD4+ T cells, and CD8+ T cells may alter innate immunity. The delay in IFN-gamma response, prolonged hyperinflammatory state, and lower CD4 and CD8 cell numbers may exacerbate the cytokine storm and therefore increase the severity of COVID-19 infection and increase the risk of fungal infections too [21,23]. The same mechanism justifies the increased rates of secondary bacterial (32.5%) and fungal coinfections (25.2%) in COVID-19 patients reported in other studies [25], as well as the 21.8% secondary bacterial infection rate in CAM, observed in another study [26].

This study has certain limitations including a small sample size of the study and a lack of histopathological and radiological correlation of the patient with these microbiological test data.

## Conclusions

The incidence of mucormycosis and other fungal coinfections has significantly increased in post-COVID-19 patients, particularly those with uncontrolled diabetes, immunocompromised states, and steroid therapy and those living in unhygienic conditions. The strict monitoring of the judicious use of antimicrobial drugs, early screening for fungal infections in suspected patients, and the control of associated risk factors are crucial in preventing and reducing the overall morbidity of COVID-19-associated mucormycosis and COVID-19-associated aspergillosis.
